# Investigation of multidirectional toxicity induced by high-dose molybdenum exposure with *Allium* test

**DOI:** 10.1038/s41598-024-59335-6

**Published:** 2024-04-15

**Authors:** Burak Özkan, Kültiğin Çavuşoğlu, Emine Yalçin, Ali Acar

**Affiliations:** 1 Department of Biology, Institute of Science, Giresun University, Giresun, Turkey; 2 Department of Biology, Faculty of Science and Art, Giresun University, 28200 Giresun, Turkey; 3 Department of Medical Services and Techniques, Vocational School of Health Services, Giresun University, Giresun, Turkey

**Keywords:** Antioxidant enzymes, Deep neural network, Genotoxicity, Lipid peroxidation, Meristematic cell damage, Physiology, Trace element, Genetics, Plant sciences

## Abstract

In this study, the multifaceted toxicity induced by high doses of the essential trace element molybdenum in *Allium cepa* L. was investigated. Germination, root elongation, weight gain, mitotic index (MI), micronucleus (MN), chromosomal abnormalities (CAs), Comet assay, malondialdehyde (MDA), proline, superoxide dismutase (SOD), catalase (CAT) and anatomical parameters were used as biomarkers of toxicity. In addition, detailed correlation and PCA analyzes were performed for all parameters discussed. On the other hand, this study focused on the development of a two hidden layer deep neural network (DNN) using Matlab. Four experimental groups were designed: control group bulbs were germinated in tap water and application group bulbs were germinated with 1000, 2000 and 4000 mg/L doses of molybdenum for 72 h. After germination, root tips were collected and prepared for analysis. As a result, molybdenum exposure caused a dose-dependent decrease (p < 0.05) in the investigated physiological parameter values, and an increase (p < 0.05) in the cytogenetic (except MI) and biochemical parameter values. Molybdenum exposure induced different types of CAs and various anatomical damages in root meristem cells. Comet assay results showed that the severity of DNA damage increased depending on the increasing molybdenum dose. Detailed correlation and PCA analysis results determined significant positive and negative interactions between the investigated parameters and confirmed the relationships of these parameters with molybdenum doses. It has been found that the DNN model is in close agreement with the actual data showing the accuracy of the predictions. MAE, MAPE, RMSE and R2 were used to evaluate the effectiveness of the DNN model. Collective analysis of these metrics showed that the DNN model performed well. As a result, it has been determined once again that high doses of molybdenum cause multiple toxicity in *A. cepa* and the *Allium* test is a reliable universal test for determining this toxicity. Therefore, periodic measurement of molybdenum levels in agricultural soils should be the first priority in preventing molybdenum toxicity.

## Introduction

Trace elements are minerals that are present in trace quantities and are necessary for the growth and metabolism of living organisms. There are a total of 19 trace elements categorized into three groups in organisms. Boron, cobalt, copper, iodine, iron, manganese, zinc and molybdenum are the essential trace elements found in organisms. These elements are structural components of enzymes that undertake different tasks such as the regulation of the reproductive system, the fulfillment of immune functions, the regulation of gene expression, the realization of antioxidant defense and the prevention of chronic diseases^1,2^. In addition, some trace elements provide the production and use of metabolic energy by removing or binding electrons in redox reactions. Some of them participate in the structure of the cell membrane by facilitating the binding of molecules to the receptor sites on the cell membrane^3^. The lack of trace elements seriously affects the life cycle of the organism, and their long-term deficiency can even cause the death of the organism. In case of deficiencies, the structures of enzymes deteriorate and enzyme function loss occurs. Trace element deficiency in plants reduces crop yield by causing chlorosis, especially in plant leaves, and may result in reduced plant growth by forming necrotic spots^4^. Overdose of trace element exposure can gradually lead to toxic and fatal consequences for organisms^5^. The increase in trace element amounts may cause the blocking of functional enzyme groups and toxicity by replacing the basic metal ion in protein-structured biomolecules^6^.

Molybdenum is an essential trace element that occurs naturally and is commonly found in nature. Molybdenum has an atomic number of 42 and an atomic weight of 95.94 and is classified as a transition metal within the class 5-B of the periodic table. It is a metal that has a metallic luster and exists in the form of a dark gray or black powder or a silvery-white mass. It is not naturally found in pure metallic form. It occurs as oxide or sulfur compounds such as powellite, molybdenite, wulfenite, ilsemannite, and ferrimolybdite. The molybdenum forms commonly found in the environment are molybdenum trioxide, ammonium molybdate, and sodium molybdate^7^. Molybdenum is the most used metal in alloy and stainless steel manufacturing. It is preferred due to it provides strength, hardness, toughness to alloys and increases temperature resistance and corrosion resistance^8^. It is also widely used in metallurgical applications. It is used as a support wire for the tungsten filament in lamps, a structural component in wind turbines, solar panels and steel manufacturing. Some of the products in which molybdenum is used are metal and magnet alloys, electronic materials, ship shafts, aircraft parts, rifle barrels, petroleum products, superheaters, fabric dyes, oil and greases, needles, crucibles, and spacecraft. Wastes from molybdenum mining activities can significantly increase the concentration of molybdenum in the environment and cause pollution in agricultural areas^7^.

Molybdenum is absolutely essential as a micronutrient for humans, animals and plants. It is also a necessary trace element for various metalloenzymes and metabolic reactions in which these enzymes take part. For example, molybdenum acts as cofactor for xanthine oxidase, aldehyde oxidase, sulfide oxidase, and mitochondrial amidoxime reducing component. For plants, it exists as a cofactor in the active center of enzymes that catalyze the basic steps of nitrogen, carbon and sulfur metabolism, and ensures efficient growth of plants under different environmental conditions. In addition, leguminous plants need molybdenum for nitrogen fixation. On the other hand, it also functions in copper and iron metabolism. It also contributes to the processes of energy production, signal transmission and waste processing in cells^9–11^. Molybdenum is absorbed into the human body from food sources. Since it is found in abundance in vegetables, fruits, legumes (beans, peas, lentils), cereals, soy products and animal products (liver, kidney), deficiency in the body is not experienced too much^10^. However, in case of deficiency, problems such as loss or reduction of molybdenum-dependent enzyme activity, mouth and gingival disorders and impotence may occur in humans^12^. Molybdenum deficiency in plants inhibits plant growth. This inhibition is due to the reduced activities of molybdenum-dependent enzymes (such as nitrate reductase). Since nitrate assimilation cannot occur in molybdenum deficiency, nitrate accumulation occurs in plants and yellowish spots and drying are observed between the veins of old leaves. In addition, uneven growth can be seen in leaves^13^. Intake of excessive amounts of molybdenum can cause seizures, hallucinations, weakened bone structure, reduced fertility and brain damage in humans. In addition, people living in geographical areas with high amounts of molybdenum in their soil and thus in the plant structure are more likely to suffer from gout. Excessive dietary intake of molybdenum also causes copper deficiency, especially in ruminant animals^11,14^. On the other hand, excess molybdenum negatively affects plant growth and decreases biomass due to the decrease in photosynthesis^15^.

Normal molybdenum concentrations in edible crops are reported to be between 0.8 and 5 mg/kg, although they vary by plant species. This level is especially higher in crops grown in areas close to mining areas. Molybdenum concentrations between 0.58 and 12.04 mg/kg were detected in rice grains grown in the agricultural area close to the molybdenum mine area. Molybdenum poisoning, which can be fatal, is also reported in animals fed with grass with high molybdenum concentrations. The presence of high concentrations of molybdenum may also occur as a result of accumulation, and therefore the effects of high molybdenum levels, especially in crop plants, should be carefully considered^16^. It is seen that studies on molybdenum in the literature mostly focus on the effects of molybdenum deficiency in organisms. The number of studies dealing with the effects of excess from molybdenum in organisms is quite insufficient. The studies carried out focused more on a single parameter. Therefore, it is of great importance to increase the number of studies in this context. In this study, the multifaceted toxicity caused by high molybdenum doses in *A. cepa* was investigated extensively with the help of multiple parameters. *Allium* test is considered a highly effective biotest for monitoring mutagenic effects. Cytotoxicity tests using in vivo plant test systems such as *A. cepa* have been validated by many researchers working with in vitro animal organism tests, and the results obtained are reported to be similar and provide important information for human health^17,18^.

## Materials and methods

### Biological indicator material

*Allium cepa* bulbs, an indicator plant, were used for experimental procedures. The bulbs were bought from a store that sold them in Giresun province in Turkey. Experimental research on plant samples, including the supply of plant material, complies with institutional, national and international guidelines and legislation.

### Chemicals and dose preference

Sodium molybdate dihydrate (CAS no: 10102-40-6-M1651) and other chemicals used were obtained from Merck company.

### Group formation and experimental principle

**Figure 1:**
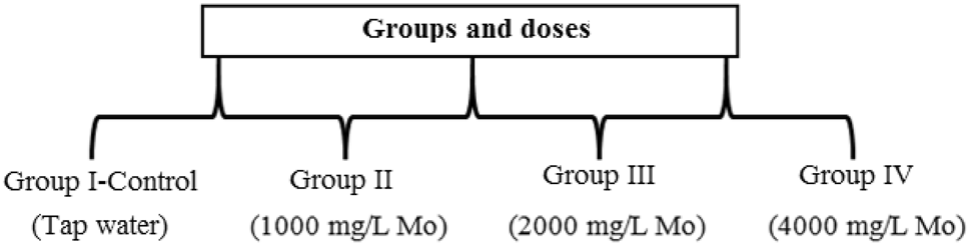
Groups and application doses.

As shown in Fig. 1, a 4 groups were formed from *A. cepa* bulbs.

The bulbs in Group I were germinated in glass containers using tap water, while the bulbs in Group II, III, and IV were germinated with sodium molybdate dihydrate in doses of 1000 mg/L, 2000 mg/L, and 4000 mg/L respectively. Doses of molybdenum were based on toxic dose ranges used in the study of Shi et al.^16^. The germination process was conducted at room temperature for a continuous period of 72 h. Following the germination process, the root tips of the bulbs were washed in distilled water before being prepared for analysis using routine preparation techniques.

### Physiological measurements

The measurements of the physiological parameters were conducted in accordance with the following criteria as identified by Aydın et al.^19^.

- The root elongation was determined by measuring the radicle length, which is responsible for root formation, using a millimeter ruler.
- Weight gain the live weights of the bulbs were determined by weighing them with precision scales before and immediately after germination (g).
- Eq. (1) was used to determine the germination.
  1 $${\text{Germination }}\left( \% \right) \, = \, \left[ {{\text{germinated }}\;{\text{bulb }}\;{\text{number}}} \right] \, / \, \left[ {{\text{total}}\;{\text{ bulb}}\;{\text{ number}}} \right] \, \times { 1}00$$

### Cytogenetic analyzes

Observation of MN and chromosomal damage was performed using Clarke fixator, according to the method suggested by Macar et al.^20^.

CAs and MN counts were carried out by 2 different researchers who are experts in the field.

The methods used to detect MN were based on the following criteria of Fenech et al.^21^.

- The diameter of the MN should be about one third of the diameter of the nucleus.
- MN should take on the same color with the nucleus.
- In the event of an interaction between the MN and the nuclear membrane, the boundary should be clearly defined.

MI has been calculated using Eq. (2).

2 $${\text{MI }}\left( \% \right) \, = \, \left[ {{\text{cell }}\;{\text{number }}\;{\text{in }}\;{\text{mitosis}}} \right] \, / \, \left[ {{\text{total}}\;{\text{ cell }}\;{\text{number}}} \right] \, \times { 1}00$$

### Comet assay

The DNA extraction from the root cells was conducted in accordance to Sharma et al.^22^ and Comet assays were performed according to the method recommended by Dikilitaş and Koçyiğit^23^. Slides prepared prior to analysis were stained with 100 µL of ethidium-bromide and analyzed under fluorescence microscopy and photographed by performing counting processes. Analyzes were performed in the TriTek 2.0.0.38 Automatic Comet Assay software program. In the comet assay analysis, the extent of DNA damage was evaluated in two ways. Image-based DNA damage level was determined according to the criteria of Jayawardene et al.^24^, and according to numerical results, criteria of Pereira et al.^25^ were used to determine the level of DNA damage.

### Biochemical measurements

#### MDA measurement

The Measurement of MDA levels was conducted in accordance with the methodology proposed by the Unyayar et al.^26^ using trichloroacetic acid and thiobarbituric acid. The supernatant absorbance was measured at a wavelength of 532 nm. Measurements were repeated three times for each group.

#### Proline measurement

The free proline content was quantified in accordance with the Bates et al.^27^ using sulfosalicylic acid, acidninhydrin, glacial acetic acid and toluene. The absorbance was measured at 520 nm. Measurements were repeated three times for each group. Proline concentration was determined from a standard curve and calculated according to Eq. (3).

3 $$\left[ {\left( {\upmu {\text{g}}\;{\text{ proline}}/{\text{mL }} \times {\text{ mL }}\;{\text{toluen}}} \right) \, /{ 115}.{5 }\upmu {\text{g}}/\upmu {\text{mol}}} \right]/\left[ {\left( {{\text{g }}\;{\text{sample}}} \right)/{5}} \right] \, = \, \upmu {\text{mol }}\;{\text{proline}}/{\text{g}}\;{\text{ of}}\;{\text{ FW }}\;{\text{sample}}$$

#### Activity measurements of antioxidant enzymes

The root tips were rinsed with distilled water and homogenised in a cool sodium phosphate buffer solution, centrifuged, and kept at + 4 °C until they were ready for analysis^28^. The SOD enzyme activity was quantified in accordance with the methodology proposed by Beauchamp and Fridovich^29^, by preparing a reaction solution and following the reaction process carried out under fluorescent lamp. The absorbance was measured at 560 nm^28^. Measurements were repeated three times for each group. The CAT enzyme activity was determined in accordance with the Beers and Sizer^30^ methodology by preparing a reaction mixture. The determination of CAT activity was based on the observation of the reduction in absorption at 240 nm in UV–VIS spectrophotometer as a result of H_2_O_2_ consumption^28^. Measurements were repeated three times for each group.

### Anatomical observations

Root tips were placed between styrofoam and semi-thin sections were taken using a razor blade. The sections taken on the slide were painted with methylene blue, covered with a coverslip, examined under a research microscope and photographed^31^.

### Deep neural network (DNN) modeling

**Figure 2:**
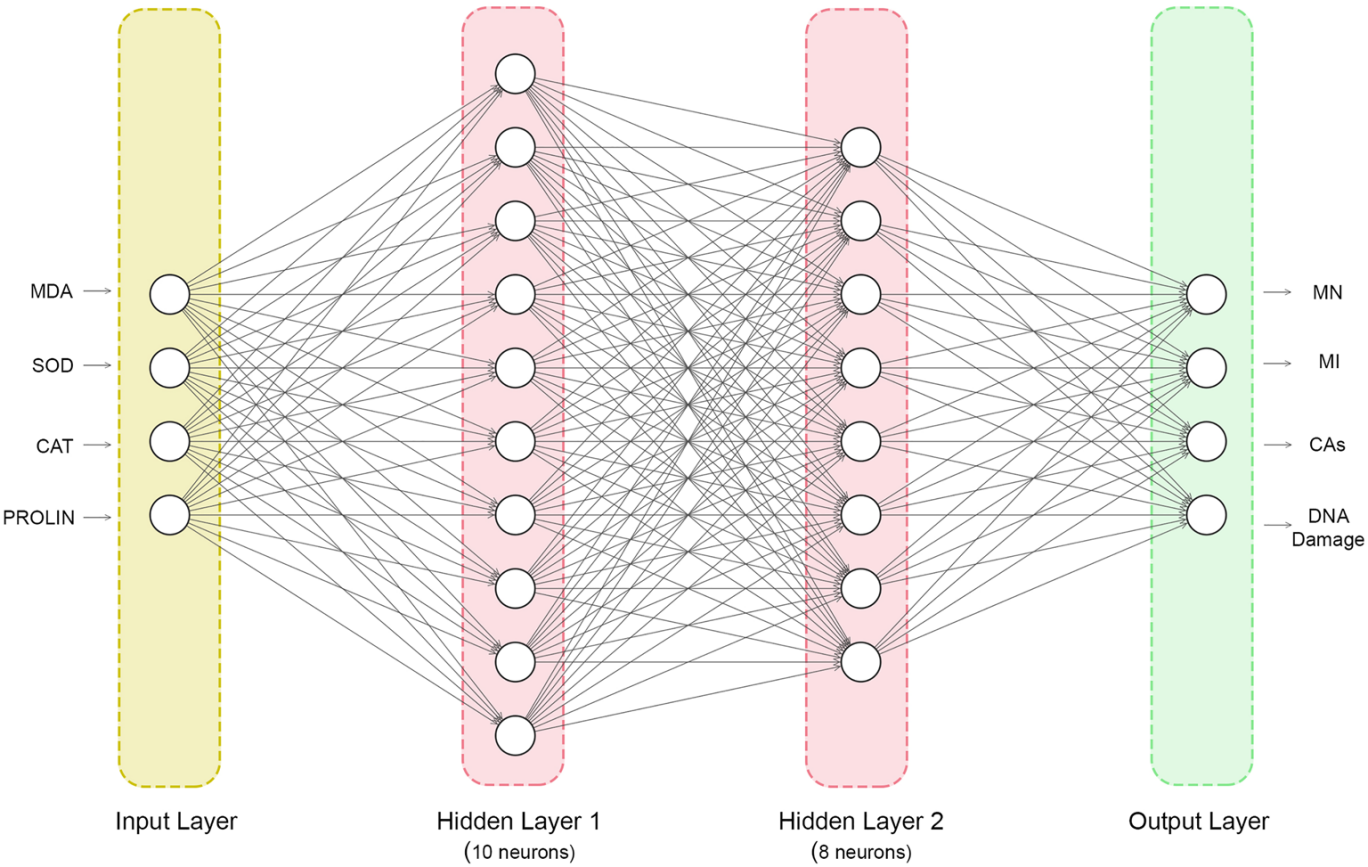
Architecture of trained deep neural network featuring two hidden layers.

The deep neural network (DNN) represents an advanced form of machine learning, characterized by multiple layers situated between the input and output layers. These layers consist of neurons that serve as the fundamental operational units, playing a crucial role in the network's task^32^. To investigate the relationship of genotoxicity with proline, lipid peroxidation, oxidative stress parameters, a feed-forward artificial neural network was employed, and the back-propagation of error algorithm was utilized. Empirical data were used to train the model, and the weights were fine-tuned to minimize the root-mean-square error (RMSE). The neural network architecture, as depicted in Fig. 2, consisted of two hidden layers and four input parameters representing proline levels, MDA levels, SOD, and CAT activities resulting from molibden application. The model had 4 output parameters corresponding to MN, MI, CAs, and DNA damage. The neural network architecture was implemented using MATLAB® R2019b (Matlab 9.7.0.1190202, Mathworks Inc, USA), with one input neuron and two hidden layers containing ten and eight neurons, respectively. To ensure effective training, the input and target data were normalized within the range of [− 1, 1] using the “mapminmax” function. The Levenberg–Marquardt back-propagation algorithm was employed for model optimization. The dataset was divided into three parts, with 70% allocated for training, 15% for testing, and 15% for cross-validation. Ultimately, the trained DNN model was utilized to predict actual data and compared against the predicted data to evaluate its performance.

### Evaluation of DNN

The accuracy of a DNN model is evaluated based on how close the predicted values are to the actual observed values. A relatively small prediction error indicates the effectiveness of the model in predicting the actual values. This study employed various error performance methods, namely mean absolute error (MAE) [Eq. (4)], mean absolute percentage error (MAPE) [Eq. (5)], root mean squared error (RMSE) [Eq. (6)] and coefficient of determination (R2) [Eq. (7)], to analyze the techniques utilized.

${y}_{i}$ is the actual data, ${x}_{i}$ is the predicted data, and $\overline{y }$ is the mean actual data;

4 $${\text{MAE}}=\sum_{{\text{i}}=1}^{{\text{N}}}|{{\text{y}}}_{{\text{i}}}-{{\text{x}}}_{{\text{i}}}|$$

5 $$\mathrm{MAPE }(\mathrm{\%})=\frac{100}{{\text{N}}}\sum_{{\text{i}}=1}^{{\text{N}}}\left|\frac{{{\text{y}}}_{{\text{i}}}-{{\text{x}}}_{{\text{i}}}}{{{\text{y}}}_{{\text{i}}}}\right|$$

6 $${\text{RMSE}}=\sqrt{\frac{1}{{\text{N}}}\sum_{{\text{i}}=1}^{{\text{N}}}{\left({{\text{y}}}_{{\text{i}}}-{{\text{x}}}_{{\text{i}}}\right)}^{2}}$$

7 $${{\text{R}}}^{2}=1-\frac{\sum_{i=1}^{N}({{y}_{i}-{x}_{i})}^{2}}{\sum_{i=1}^{n}{\left({y}_{i}-\overline{y }\right)}^{2}}$$

### Statistical evaluation

The data was analyzed using SPSS package program in IBM Statistics 22. Statistical significance was investigated using the “One-way Anova” and “Duncan” tests. If the p value obtained as a result of the analysis was less than 0.05, it was considered statistically significant.

Correlation and principal component analysis (PCA) were conducted utilizing Rstudio v1.4.1106^33^. Pearson correlation analysis (two-sided) and visualizations were generated using the Hmisc and corrplot packages^34^. PCA was carried out to investigate various toxicity markers encompassing physiological, cytogenetic, and biochemical parameters. PCA was executed with the aid of the factoMineR^35^ and factoextra^36^ packages, which are accessible within the RStudio environment.

## Results and discussion

### Physiological findings

Table 1 shows the physiological toxicity of molybdenum. The highest physiological parameter values were observed in the control group (GI). In this group, 100% germination rate, mean 12.5 cm root length and mean 8.40 g weight gain were determined. Molybdenum exposure caused remarkable decreases (p < 0.05) in all physiological parameter values. It was determined that this decrease was dependent on the applied molybdenum dose. Among the groups exposed to molybdenum, the highest decrease in all physiological parameter values was measured in GIV, which was exposed to a dose of 4000 mg/L of molybdenum. Compared to the control group, germination decreased by 24%, root length decreased approximately 1.9 times, and weight gain decreased approximately 2.4 times in GIV. These decreases were found to be statistically significant (p < 0.05).

**Table 1:** Effect of molybdenum on bulb physiology.

Groups	Germination percent (%) (n = 50)	Root length (cm) ± SD (n = 10)	Weight gain (g) (n = 10)	Initial weight (g) ± SD	Final weight (g) ± SD
GI	100	12.5 ± 1.00^a^	+ 8.40^a^	12.6 ± 1.15	21.0 ± 1.33
GII	93	11.0 ± 0.89^b^	+ 7.00^b^	12.5 ± 1.19	19.5 ± 0.86
GIII	85	8.80 ± 0.74^c^	+ 5.30^c^	12.7 ± 1.19	18.0 ± 0.90
GIV	76	6.50 ± 0.80^d^	+ 3.50^d^	12.6 ± 1.18	16.1 ± 0.80

GI: Control, GII: 1000 mg/L molybdenum, GIII: 2000 mg/L molybdenum, GIV: 4000 mg/L molybdenum. Statistical significance (p < 0.05) is indicated by the letters (a) to (d) displayed along the column.

Our findings are in line with the results of the few studies investigating the physiological effects of molybdenum toxicity in plants. Bodi et al.^37^ reported that molybdenum stimulated growth up to 30 mg/kg in seedlings of *Zea mays* L. (corn) and *Helianthus annuus* L. (sunflower) grown in soil containing molybdenum at 30, 90 and 270 mg/kg doses, while at other doses it caused a decrease in shoot and root dry weights and growth. Gopal et al.^38^ observed that low-dose molybdenum caused intervascular chlorosis in young and middle leaves in *Vigna mungo* L. (black lentil) plants grown in refined sand containing molybdenum at a dose range of 0.002 μM to 2 μM for 70 days. They also found that high dose molybdenum caused a decrease in total dry matter, seed protein levels, seed yield and germination potential. Sharma et al.^39^ determined that molybdenum trioxide (MoO_3_) exposure at 100 ppm, 500 ppm, and 1000 ppm doses caused a gradual decrease in root and shoot lengths of *Oryza sativa* L. (rice) seedlings. Aragão et al.^40^ reported that environmental wastes containing trace elements such as Cu, Fe, Mn and Zn significantly reduced root growth in *Allium cepa* and *Lactuca sativa* during germination.

The decrease observed in physiological parameter values as a result of excessive molybdenum exposure can be explained by the fact that molybdenum prevents micro and macro element uptake by *A. cepa* roots, causes damage to the anatomical structure of the roots and reduces the mitotic division of root cells. Because studies in the literature indicating that high amounts of trace elements cause physiological toxicity support our idea. For example, exposure to excessive amounts of trace elements such as copper^41^ and cobalt^42^ has been reported to block the uptake of water and nutrients of *A. cepa* roots. Gopal et al.^38^ determined that excessive molybdenum exposure damaged the physiological structure of plants by causing a decrease in starch, sugar, protein and nitrogen contents. Vachirapatama et al.^43^ observed that high-dose vanadium exposure damaged the root structure of *Solanum lycopersicum* L. (tomato) and prevented the roots from working efficiently. Similarly, Kalefetoğlu Macar et al.^44^ determined that exposure to cobalt at a dose of 5.5 ppm caused deformation in the epidermis and cortex cells of *A. cepa* roots and disrupted the root structure. In our study, different structural abnormalities that damage the root structure of *A. cepa* were observed. In addition, there are some studies in the literature that trace element toxicity inhibits cell division in plant root tips and reduces root elongation^45,46^. In our research, we found that when we increased the dose of Molybdenum, root meristem cell MI values went down.

### Cytogenetic findings

**Figure 3:**
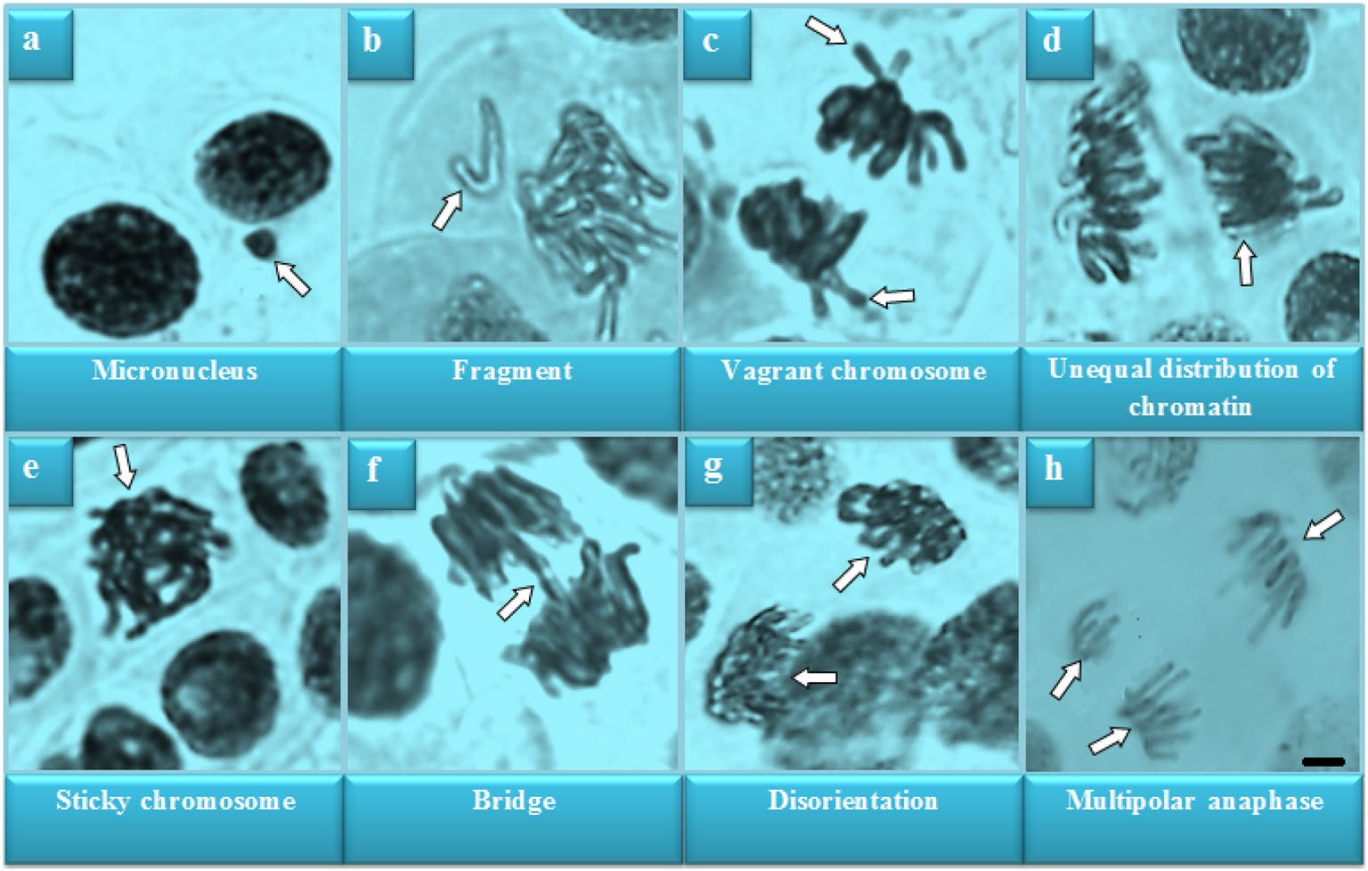
Chromosomal abnormalities induced by molybdenum (× 400). Bar: 10 µm.

**Figure 4:**
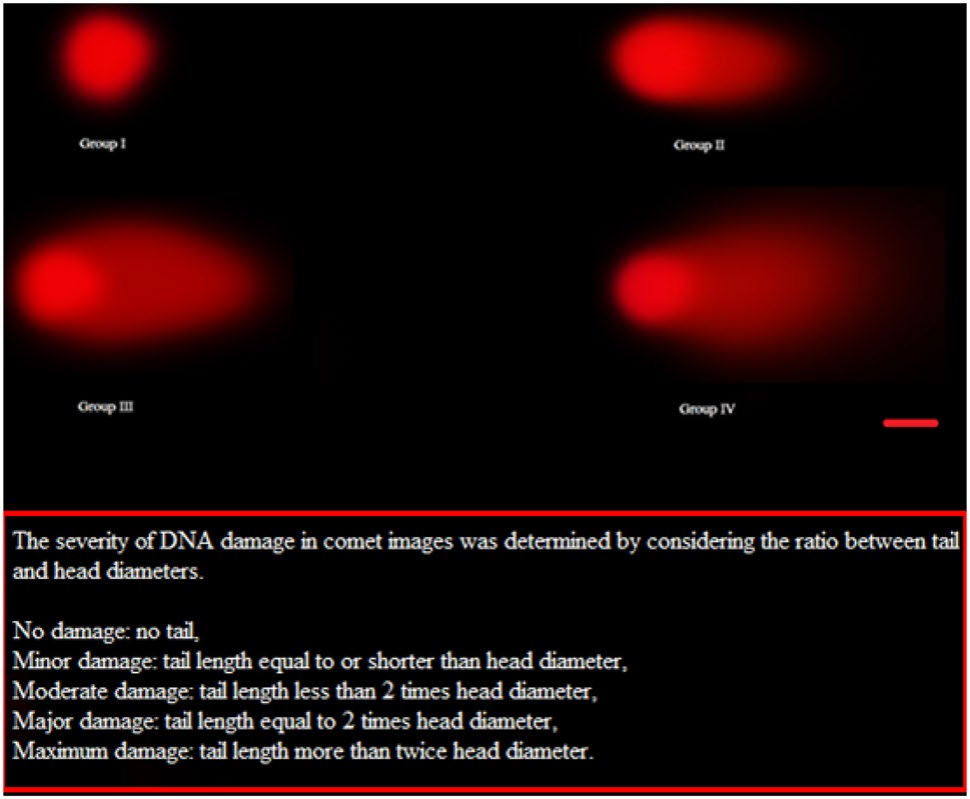
DNA damage caused by molybdenum exposure (× 400). GI: Control, GII: 1000 mg/L molybdenum, GIII: 2000 mg/L molybdenum, GIV: 4000 mg/L molybdenum. Bar: 10 µm.

Molybdenum-induced genotoxicity is illustrated in Fig. 3 and Table 2. The highest mean of MI and the lowest mean of MN and CAs were counted in the control group (GI). In this group, 8.96% MI, 0.30% MN, and only 0.20% CAs in the form of unequal distribution of chromatin were determined. Depending on the dose, molybdenum caused decreases (p < 0.05) in MI and increases (p < 0.05) in MN frequency and CAs numbers. Compared to the control group, MI was reduced by approximately 24.8% in GIV exposed to 4000 mg/L molybdenum. However, the frequency of MN increased approximately 27 times and the number of fragment, which was the most observed abnormality, increased approximately 24 times. Molybdenum exposure promoted CAs such as fragment > vagrant chromosome > unequal distribution of chromatin > sticky chromosome > bridge > disorientation > multipolar anaphase in root meristem cells. Exposure to molybdenum also resulted in a dose-dependent increase in DNA damage. The biggest effect of molybdenum exposure on chromosomes is fragment formation. Fragments and bridge abnormalities are clastogenic manifestations resulting from breaks in chromosomes and chromatids^47^. Vagrant chromosomes are chromosomes that cannot be pulled to the poles as a result of spindle damage. Sticky chromosomes can result from defects in nucleic acid metabolism, adhesion of chromosomal proteins, or dissolution of DNA-related proteins. Also, Himtaş et al.^48^ reported that chromosomal abnormalities such as sticky chromosomes are associated with the inhibition of microtubule and spindle fiber formation. Additionally, chromosome stickiness can cause lethal effects and is associated with the effects of environmental pollutants, including pesticides. Nuclei formations of unequal sizes or irregular shapes may be observed in daughter cells with unequal distribution of chromatin^49,50^. Each chromosomal abnormality observed as a result of cytogenetic analysis occurs as a result of different mechanisms. This shows that Mo application triggers the genotoxic effect through different mechanisms. Comet assay analysis results showed a decrease in head DNA percentages (p < 0.05) and an increase in tail DNA percentages depending on the molybdenum dose (Fig. 4 and Table 3).

**Table 2:** Genotoxicity caused by high dose molybdenum.

Abnormalities ± SD (n = 10)	GI	GII	GIII	GIV
Mitotic index %	896 ± 16.5^a^ (8.96)	835 ± 20.7^b^ (8.35)	752 ± 13.5^c^ (7.52)	674 ± 11.6^d^ (6.74)
Micronucleus	0.30 ± 0.48^d^	6.30 ± 1.77^c^	12.8 ± 1.69^b^	26.9 ± 2.02^a^
Fragment	0.00 ± 0.00^d^	5.80 ± 1.03^c^	10.4 ± 2.12^b^	23.7 ± 1.95^a^
Vagrant chromosome	0.00 ± 0.00^d^	5.10 ± 1.20^c^	9.80 ± 1.69^b^	21.3 ± 1.89^a^
Unequal distribution of chromatin	0.20 ± 0.42^d^	4.70 ± 1.16^c^	8.20 ± 1.40^b^	17.8 ± 1.62^a^
Sticky chromosome	0.00 ± 0.00^d^	3.90 ± 0.88^c^	7.40 ± 1.17^b^	15.6 ± 2.07^a^
Bridge	0.00 ± 0.00^d^	3.00 ± 0.82^c^	6.50 ± 1.08^b^	13.5 ± 1.27^a^
Disorientation	0.00 ± 0.00^d^	2.50 ± 1.08^c^	4.90 ± 1.52^b^	10.2 ± 1.48^a^
Multipolar anaphase	0.00 ± 0.00^d^	2.10 ± 0.88^c^	4.40 ± 0.97^b^	8.70 ± 1.34^a^

GI: Control, GII: 1000 mg/L molybdenum, GIII: 2000 mg/L molybdenum, GIV: 4000 mg/L molybdenum. 1000 cells per group for MN and CAs and 10,000 cells per group for MI were analyzed. Statistical significance (p < 0.05) is indicated by the letters (a) to (d) displayed along the line.

**Table 3:** Comet analysis data showing DNA damage.

Analysis data ± SD (n = 10)	GI	GII	GIII	GIV
Head diameter (px)	100.000	116.000	84.000	80.000
Head density	1.794.975	2.270.870	1.813.22	1.153.906
Head DNA (%)	**100 ± 0.00**^**a**^	**73.0 ± 1.76**^**b**^	**36.0 ± 2.67**^**c**^	**24.0 ± 1.70**^**d**^
Tail length (px)	1.000	86.000	229.000	292.000
Tail density	1.000	831.172	3.224.335	3.645.400
Tail DNA (%)	**0.00 ± 0.00**^**d**^	**27.0 ± 1.76**^**c**^	**64.0 ± 2.67**^**b**^	**76.0 ± 1.70**^**a**^
Tail moment	0.000001	23.043.140	146.573.959	221.793.900

Significant values are in bold.

GI: Control, GII: 1000 mg/L molybdenum, GIII: 2000 mg/L molybdenum, GIV: 4000 mg/L molybdenum. The analysis of DNA damage was conducted on 1000 cells in each of the four groups. Statistical significance (p < 0.05) is indicated by the letters (a) to (d) displayed along the line. The Comet scale for DNA damage was calculated based on the percent of tail DNA. If the tail DNA percentage is ≤ 5% no or very little damage, 5–20% low damage, 20–40% moderate damage, 40–75% high damage, ≥ 75% severe damage.

There is no comprehensive study in the literature investigating the genotoxicity caused by molybdenum or molybdenum compounds in plant cells. However, there is a limited amount of research conducted on the effects of other trace elements on genotoxicity. Güler et al.^51^ reported that 2.4, 8.0 and 12.5 mg/L doses of chromium caused a decrease in the MI value of *A. cepa* root meristem cells, and an increase in the frequency of MN and the number of CAs. Macar et al.^45^ determined that 20 µM dose of copper caused a decrease in MI and an increase in MN frequency and CAs in *A. cepa* root cells. Kalefetoğlu Macar et al.^44^ observed that 5 ppm dose of cobalt caused a decrease in MI value of *A. cepa* root meristem cells, and an increase in MN frequency and CAs numbers. Using the comet assay method, Kaya et al.^52^ showed that DNA damage scores in *A. cepa* root cells increased due to the increase in the dose of vanadium.

One reason for the genotoxicity observed in *A. cepa* root meristem cells as a result of molybdenum exposure may be the direct interaction of molybdenum with DNA or microtubules. The information in the literature that metals can interact with DNA and nuclear proteins to cause conformational changes that lead to DNA damage, cell cycle modulation and controlled cell death also supports our this idea^53^. In addition, it has been shown that copper, one of the essential trace elements, disrupts the microtubule structure in *Allium sativum* L. (garlic) root tip cells, causing tubulin aggregation, disruption in chromosome movements, preventing cells from entering mitosis, fragmentation in microtubules and microtubule depolymerization^54^. Another reason of molybdenum genotoxicity is because it helps create reactive oxygen species (ROS), which can end up damaging DNA and the structure of microtubules. The information in the literature that trace elements or heavy metals such as Pb, Cd, Al, Ni and Cr increase the production and accumulation of ROS in plants and cause damage to macromolecules by interacting with enzymes, lipids, proteins and genetic material supports our this idea^55^.

### Biochemical findings

Table 4 illustrates the biochemical toxicity associated with the molybdenum. The contol group (GI) group demonstrated the lowest levels of MDA, proline, SOD, and CAT. In this group, mean 10.4 MDA, mean 15.6 proline, mean 45.8 SOD activity and mean 1.10 CAT activity were measured. Molybdenum caused remarkable increases (p < 0.05) in all biochemical parameter values depending on the dose. These increases were highest in GIV exposed to a 4000 mg/L molybdenum dose. MDA and proline levels increased by approximately 2.5-fold, SOD activity increased by approximately 1.7-fold, and CAT activity increased by approximately 2.5-fold in GIV compared to the control group.

**Table 4:** Effects of excessive molybdenum doses on root biochemistry.

Groups	MDA (µM/g FW) ± SD (n = 10)	Proline (µmol/g FW) ± SD (n = 10)	SOD (U/mg FW) ± SD (n = 10)	CAT (OD_240 nm_ min/g FW) ± SD (n = 10)
GI	10.4 ± 1.96^d^	15.6 ± 1.71^d^	45.8 ± 1.93^d^	1.10 ± 0.14^d^
GII	14.5 ± 1.72^c^	22.7 ± 2.11^c^	52.6 ± 2.17^c^	1.68 ± 0.21^c^
GIII	19.8 ± 1.75^b^	30.2 ± 2.04^b^	63.4 ± 1.84^b^	2.18 ± 0.25^b^
GIV	25.7 ± 1.77^a^	38.9 ± 1.85^a^	77.1 ± 2.02^a^	2.78 ± 0.18^a^

GI: Control, GII: 1000 mg/L molybdenum, GIII: 2000 mg/L molybdenum, GIV: 4000 mg/L molybdenum. Statistical significance (p < 0.05) is indicated by the letters (a) to (d) displayed along the column.

Our results are in line with the results of a limited number of studies dealing with the biochemical toxicity of molybdenum in different plant species. Kumchai et al.^56^ reported an increase in APX, CAT and SOD activities in *Brassica oleracea* var. *capitata* (cabbage) seedlings exposed to excessive molybdenum stress (in the dose range of 0.1 mM–10 mM). Xu et al.^15^ found an increase in SOD, CAT, peroxidase (POD) and APX enzyme activities in the leaves and roots of soybean seedlings grown under extreme molybdenum stress. Acemi et al.^57^ determined that molybdenum exposure at doses of 2.5 mM and above caused increases in proline and MDA levels, and POD, SOD and CAT enzyme activities of *Amsonia orientalis* (blue star). Aragão et al.^40^ reported that environmental wastes containing trace elements such as Cu, Fe, Mn and Zn significantly increased SOD activity and decreased CAT activity in *Allium cepa*.

MDA is produced as a by-product of the degradation of polyunsaturated fatty acids in the cell membranes. In other words, MDA is one of the end products of lipid degradation and is used as a powerful indicator to assess cell damage^58,59^. In scientific studies, it has been reported that MDA can cause changes in the structure of DNA and RNA, promote cross-linking in macromolecules such as protein and lipid, and suppress many genes that play a role in the plant's defense against stress factors. At the same time, MDA exhibits carcinogenic and mutagenic properties^60^.

Proline (C_5_H_9_NO_2_) is one of the most important amino acids produced by plants exposed to different stress factors^61^. It is the first synthesized product as a protection response in the plant under stress. In this way, it also provides the initiation of the metabolic reaction process that activates the plant's defense mechanism against the stress factor^62^. In addition, proline is also involved in the maintenance of osmosis and turgor pressure in plants under stress. However, it stabilizes and protects the cell membrane during dehydration^63^.

One of the most well-known effects of metal toxicity is that it promotes ROS production and causes irreversible damage to DNA, lipid and protein structure in plants. Antioxidant defense system enzymes are utilized by plants to eliminate the presence of ROS. The most important of these enzymes are SOD and CAT. SOD is one of the most powerful antioxidant enzymes that the cell has. In other words, it is an important antioxidant defense enzyme in cells exposed to oxygen. It's a key part of a system designed to protect against ROS. It helps turn superoxide radical into H_2_O_2_. Superoxide radical is produced as a by-product of oxygen metabolism and if it is not neutralized, it causes serious cell damage. CAT is one of the tetrameric antioxidant enzymes that are found in all oxygen-sensitive organisms, including bacteria, plants and animals. It works in liaison with SOD in the cell. It catalyzes the decomposition of H_2_O_2_ formed by SOD into water and molecular oxygen. H_2_O_2_ is a harmful product and must be removed from the cell. CAT has the highest turnover of all enzymes. It can convert millions of H_2_O_2_ into water and oxygen per second^64^.

This study suggests that the biochemical toxicity measured in *A. cepa* root cells may be due to molybdenum promoting free radical formation in the cell. These free radicals damage the root cell membrane and destroy lipids, resulting in the formation of MDA. Root cells can increase the synthesis of SOD and CAT by activating proline production or antioxidant defense enzyme genes to reduce this destructive effect of free radicals. Some information in the literature supports this idea. Gawel et al.^58^ reported that one of the most important factors promoting the lipid peroxidation process in an organism is free radicals and that increases in the amount of free radicals cause overproduction of MDA. Kumchai et al.^56^ determined that antioxidant enzyme activity and anthocyanin accumulation increased to scavenge ROS produced in plant cells exposed to high dose molybdenum. They stated that these antioxidants induced in plants act as protection against stress conditions. Acemi et al.^57^ showed that molybdenum exposure caused loss of integrity of root cell membranes in *A. orientalis*, resulting in MDA accumulation, and increased proline synthesis was not sufficient to maintain cell membrane stability. According to Xu et al.^15^, elevated antioxidant enzyme activity in the roots and leaves of plants contributes to the tolerance of molybdenums in plants by maintaining the homeostasis of ROS.

### Anatomical findings

**Figure 5:**
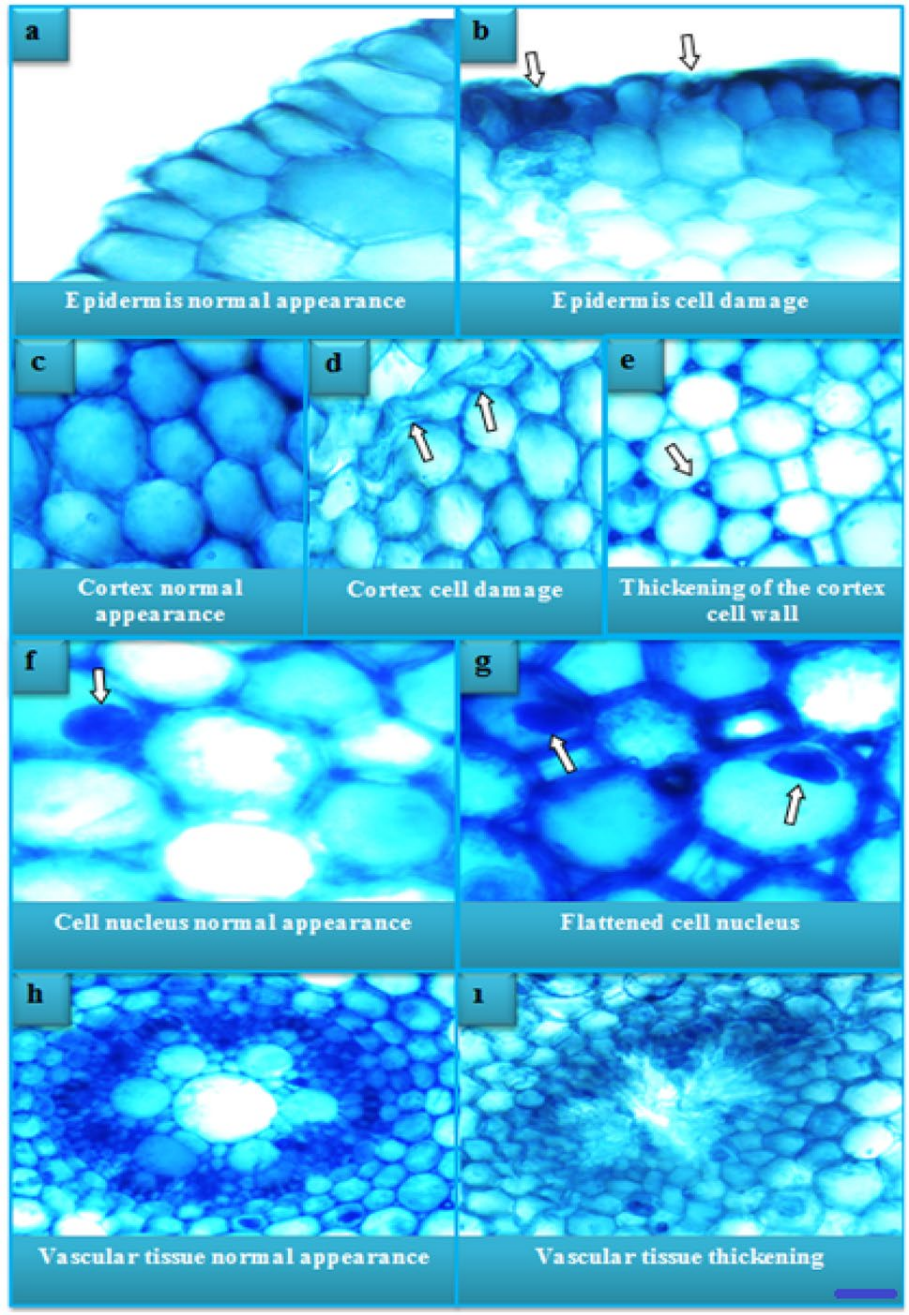
Effects of excessive molybdenum doses on root anatomy (× 200). Bar: 10 µm.

The damage and severity induced by molybdenum exposure in *A. cepa* root meristem cells are shown in Fig. 5 and Table 5. No damage was seen in the control group (GI) meristem cells under the microscope. Molybdenum exposure promoted anatomical damage such as epidermis cell damage, cortex cell damage, cortex cell wall thickening, cell nucleus flattening, and vascular tissue thickening. In addition, the severity of these damages was found to increase with the exposure to the molybdenum.

**Table 5:** Severity of anatomical damage caused by molybdenum.

Groups	Epidermis cell damage	Cortex cell damage	Cortex cell wall thickening	Flattened cell nucleus	Thickening in the vascular tissue
GI	−	−	−	−	−
GII	+	+	+	**+**	−
GIII	++	++	++	++	+
GIV	+++	+++	+++	+++	++

GI: Control, GII: 1000 mg/L molybdenum, GIII: 2000 mg/L molybdenum, GIV: 4000 mg/L molybdenum. (−): no damage, (+): little damage, (++): moderate damage, (+++): severe damage.

While there are no comprehensive studies in the literature on the anatomical alteration of plant roots caused by molybdenum, there are several studies on the alteration caused by other trace elements. Macar et al.^42^ reported that exposure to 5.5 mg/kg dose of cobalt caused damage to *A. cepa* root cells such as epidermis cell damage, flattened cell nucleus and thickening of the cortex cell wall. Kalefetoğlu Macar et al.^41^ determined that copper exposure at a dose of 20 µM caused damage such as epidermis cell damage, flattened cell nucleus, binuclear cell and cortex cell wall thickening in the meristem cells of *A. cepa* roots. Tümer et al.^65^ observed that exposure to manganese at 250, 500 and 1000 µM doses caused damage to *A. cepa* root meristem cells such as epidermis cell damage, cortex cell damage, flattened cell nucleus and cortex cell wall thickening. They also found that the damage got worse as the dose of manganese went up.

It suggests that the epidermis and cortex cell damages and flattened cell nucleus damage observed in root meristem cells as a result of molybdenum exposure may be due to the mechanical pressure formed as a result of the physical defense mechanisms developed by the root cells to avoid taking in excess molybdenum. Because in studies conducted under a microscope, a significant increase in the amounts of epidermis and cortex cells was observed in the group IV exposed to the highest molybdenum concentration compared to the control group. These cellular increases, which are done to stop the accumulation of moly bdenum in the cell, also cause the cells to come into contact with each other, resulting in a mechanical stress. This stress can lead to deformities in epidermis and cortex cells, as well as their nuclei. Damages in the form of cortex cell wall and conduction tissue thickening are thought to occur due to morphological barrier mechanisms developed to reduce the effect of molybdenum entering the cell. The information that plants develop various chemical, physical and morphological defense mechanisms to protect against metal toxicity and reduce its effects supports our idea^65–67^. Another reason for the deformation in root cells may be the deterioration of cell membrane structures. Because when molybdenum is absorbed by the roots, the first affected tissues are the epidermis and cortex tissues. With the effect of molybdenum, lipid destruction (peroxidation) can be seen in these tissues. As a result, cell deformation may occur due to disruption of membrane structures. On the other hand, the entry of molybdenum into the cell may cause a change in intracellular pressure, promoting flattening of the cell nucleus. In addition, the changes in cell nuclear volume and shape may be caused by changes in DNA structure, DNA volume and nuclear protein concentration^68,69^.

### Correlation and principal component analysis findings

**Figure 6:**
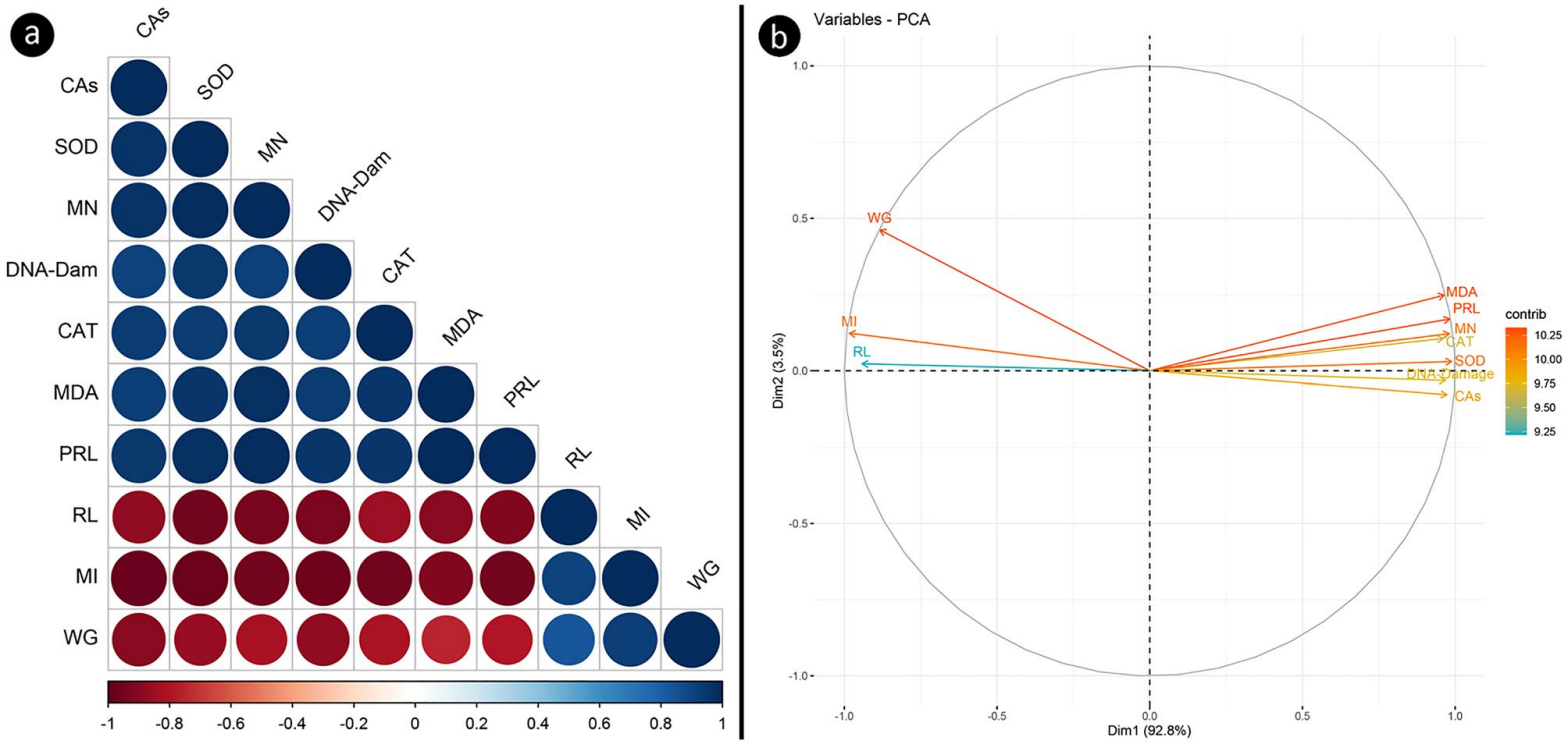
Correlation and principal component analyses of all chosen parameters. Correlation analysis (**a**), principal component analysis (**b**).

Correlation analyzes of the determined parameters are shown in Fig. 6a. Spheres in blue and shades represent positive correlations, and spheres in shades of red represent negative correlations. The hues shown and the diameter of the spheres correspond to the size of the correlation coefficients. Correlation analysis revealed significant positive or negative interdependencies between each of the selected parameters. MDA level, proline (PRL) level, SOD and CAT activities showed negative correlations with weight gain (WG), root length (RL) and MI, while positive correlations with DNA damage, MN frequency and CAs number. These findings highlight the direct influence of the examined parameters on each other.

PCA showing clustering of selected toxicity biomarkers is shown in Fig. 6b. PCA has enabled a more accurate and comprehensive assessment of the state of toxicity and the correlation between biomarkers. The analysis focused on investigating the relationships between two physiological, 4 biochemical and 4 genetic parameters. The biplot in Fig. 6b revealed that the first axis (dim1) contributed to 92.8% of the variance, while the second axis (dim2) accounted for the majority of the variance at 3.5%. Together, these two dimensions represented 96.3% of the overall variance. The analysis results demonstrated that MDA, proline, MN, CAT, SOD, DNA damage, and CAs components were closely positioned along the dim1 axis, displaying highly positive correlations. Moreover, DNA damage and CAs exhibited slightly negative components along the dim2 axis, whereas the other parameters displayed slightly positive components. On the other hand, WG, MI, and RL showed highly negative components along the dim1 axis, while WG demonstrated moderately positive components, and the remaining parameters displayed slightly positive components along the dim2 axis. The proximity of parameters in the graph indicated a stronger mutual influence, while parameters in opposing positions exhibited negative interactions, and the strength of this interaction varied based on the angle between the parameters. The outcomes of the PCA reaffirmed the interconnectedness of all selected parameters.

### Deep neural network (DNN) findings

**Figure 7:**
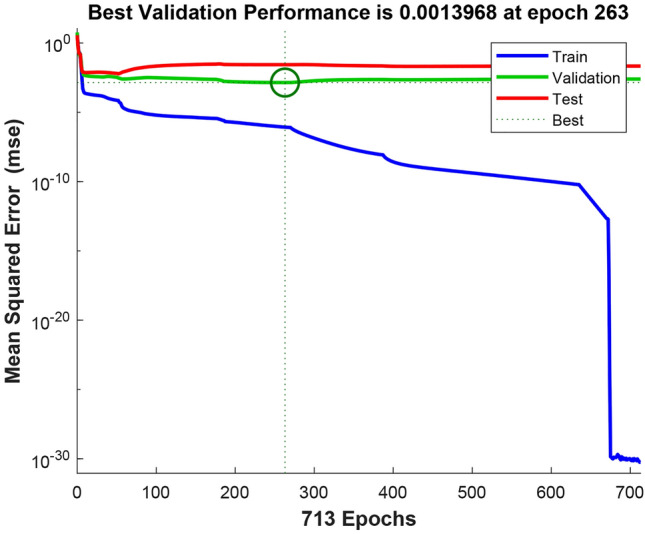
The optimal performance achieved by the DNN during the training process.

**Figure 8:**
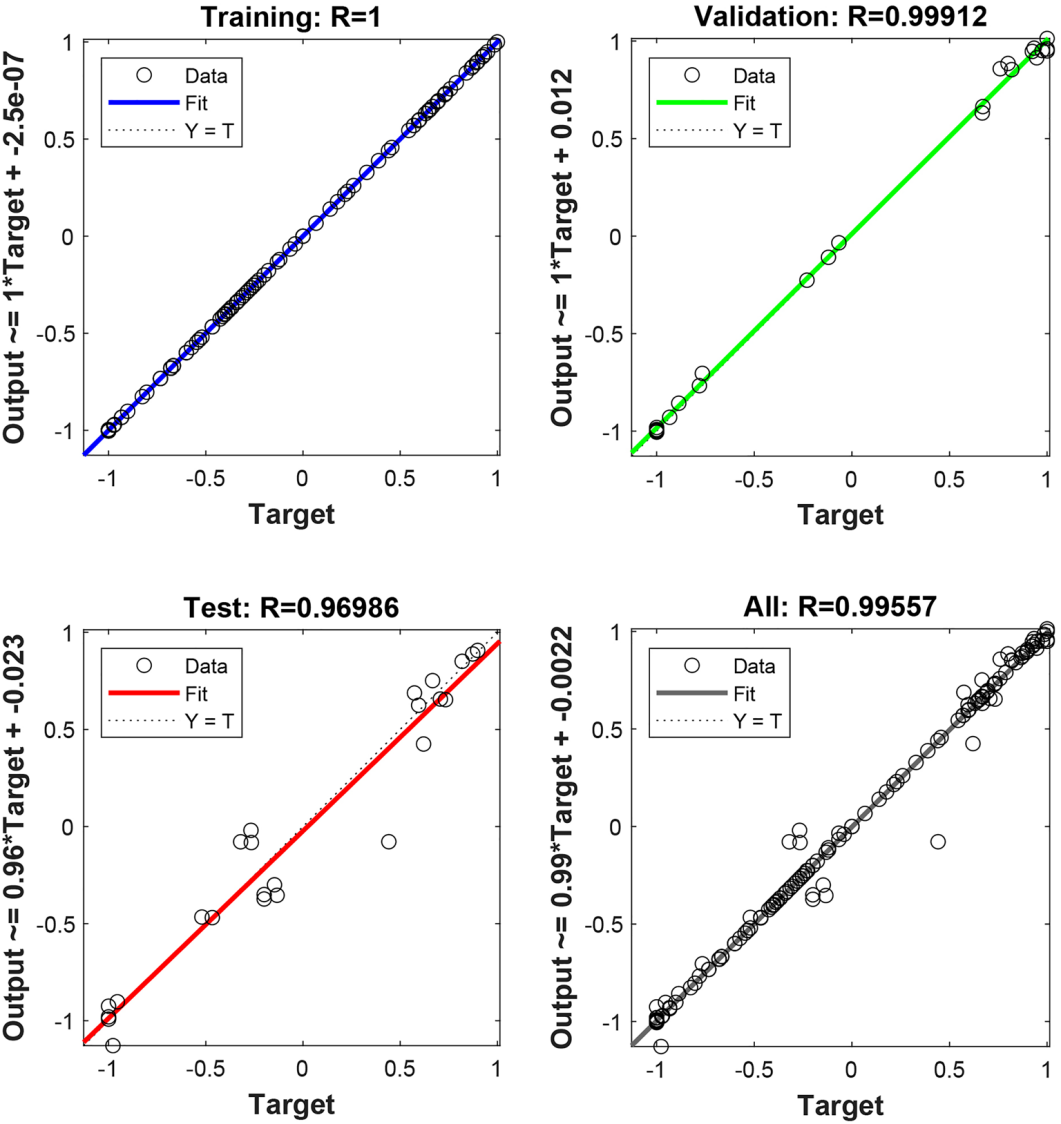
R values of the dataset for the trained network.

In this study, a multilayer perceptron regression model was used to predict biomarkers associated with genotoxicity. The model underwent fine-tuning, utilizing the tangent sigmoid function (tansig) as the activation function and adopting the RMSE as the loss function. Remarkably, at epoch 263, the model demonstrated its highest validation performance, attaining a minimal mean squared error value of 0.0013968 (Fig. 7). The analysis of the model's datasets presented in Fig. 8 demonstrates strong correlation coefficients, surpassing 0.9 for all datasets, including training, testing, validation, and the overall model. The model's performance was assessed with optimal R values: 1 for training, 0.99912 for validation, 0.96986 for testing, and 0.99557 for all datasets.

**Figure 9:**
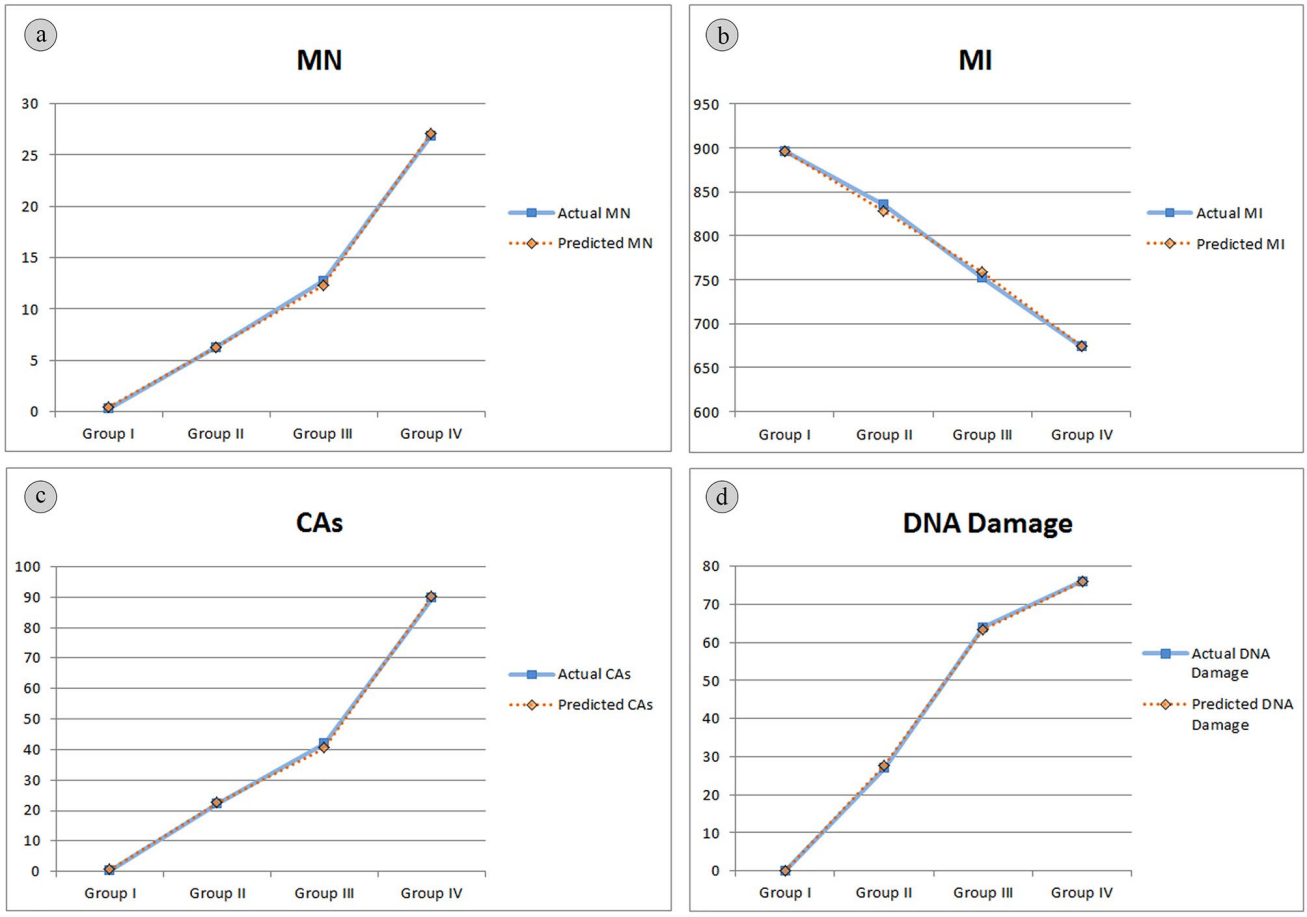
Comparison of means of actual and predicted data of dose-related molibden genotoxicity. MN (**a**), MI (**b**), CAs (**c**), DNA damage (**d**).

The DNN used in this study was trained with the Levenberg–Marquardt backpropagation algorithm, which includes four input variables (proline and MDA level, SOD and CAT activity) and four output variables (MN, MI, CAs and DNA core parameters). Figure 9 visually presents the actual data alongside the predicted data, revealing a close alignment between the two datasets, signifying the DNN model's proficient performance in forecasting genotoxicity biomarkers. The predictions of the model closely matched the real data, once again confirming its accuracy. The performanca of DNN model was comprehensively assessed through various performance metrics, including MAE, MAPE, RMSE, and R^2^. The MAE gauges the average absolute difference between predicted and actual values, while the MAPE, a percentage version of MAE, quantifies the error as a percentage of the actual value. The RMSE measures the standard deviation of the differences between predicted and actual values, while the R^2^ indicates the proportion of variance in the dependent variable (genotoxicity biomarkers) that can be predicted from the independent variables (proline and MDA levels, SOD and CAT activities). The DNN model achieved an MAE of 1.6564, signifying that its predictions were, on average, approximately 1.66 units away from the actual values. The MAPE of 11.1387 suggests that, on average, the model's predictions had a percentage error of around 11.14%. Furthermore, the RMSE of 6.9495 indicates that, on average, the model's predictions were approximately 6.95 units away from the actual values. The R^2^ value of 0.9996 indicates that an impressive 99.96% of the variability in the dependent variable (genotoxicity biomarkers) can be attributed to the independent variables (proline and MDA levels, SOD and CAT activities). Collectively, the performance metrics signify the exceptional performance of the DNN model in predicting genotoxicity biomarkers based on proline level, lipid peroxidatiomn and oxidative stress parameters. The strong correlation observed between actual and predicted values underscores the robustness and reliability of the DNN model. This study stands as the first to predict genotoxicity using neural networks, incorporating lipid peroxidation and oxidative stress parameters based on molibden application. Likewise, existing literature indicates that artificial and deep neural networks have demonstrated suitability as models for handling oxidative stress, lipid peroxidation, and antioxidant enzymes^70^, as well as environmental pollution data^71,72^.

## Conclusion

It has been determined that exposure to high doses of the trace element molybdenum causes multiple toxicity in *A. cepa*, promoting different types of chromosomal and anatomical damage and DNA damage. The *Allium* test has once again been confirmed as a robust universal test for the determination of this toxicity. Only a small amount of research has been done on the toxic effects of trace element molybdenum in the literature. Existing studies have focused more on a single parameter (physiological or biochemical). This is the most comprehensive study dealing with all aspects of molybdenum toxicity. It is also the first study to comprehensively address the cytogenetic and anatomical effects of molybdenum in plants. For this reason, it will be able to contribute a lot to the literature. Today, molybdenum spreads to the environment especially due to mining activities and causes molybdenum accumulation in agricultural areas. Molybdenum, which accumulates excessively in the soil, is added to the plant structure and can reach humans and animals from there through the food chain. For this reason, periodic measurement of molybdenum levels in agricultural production soils may be one of the most important measures to prevent molybdenum toxicity.

On the other hand, the effectiveness of the DNN model developed in this study in accurately predicting proline and MDA levels and the values of MN, MI, CAs and DNA damage based on SOD and CAT activities was emphasized. Successful establishment of this DNN model could have important implications in the field of environmental toxicology, providing a reliable and effective tool to evaluate the genotoxic effects of molybdenum. The potential of this model extends to predicting genotoxicity at different molybdenum doses and provides valuable information on selected genotoxicity biomarkers. Such information can be useful for regulatory agencies in formulating guidelines for the safe use of molybdenum. By estimating potential genotoxicity at various doses, regulatory agencies can establish maximum safety thresholds and ensure the safety and well-being of the public.

## Data Availability

The datasets used and/or analyzed during the current study are available from the corresponding author on reasonable request.
